# Vitamin D deficiency and multiple sclerosis relapse: a meta-analysis

**DOI:** 10.3389/fneur.2025.1727615

**Published:** 2026-01-02

**Authors:** Yujing Zhang, Shasha Yu, Yu Zu, Jing Lv, Jianhua Chen, Xuedan Feng

**Affiliations:** Department of Neurology, Bejing Fengtai You’anmen Hospital, Beijing, China

**Keywords:** meta-analysis, multiple sclerosis, relapse rate, systematic review, vitamin D

## Abstract

**Objective:**

This meta-analysis was conducted to systematically evaluate the dose–response relationship between serum 25-hydroxyvitamin D [25(OH)D] levels and the annualized relapse rate (ARR) in multiple sclerosis (MS), and to investigate the effect of vitamin D supplementation on reducing relapse risk.

**Methods:**

Randomized controlled trials (RCTs) published from inception to April 2025 were retrieved from PubMed, Medline, Web of Science, Cochrane Library, and EMBASE. Study quality was assessed using the Cochrane RoB-2 tool. Meta-analyses were performed using RevMan 5.3. The primary outcome was ARR. Secondary outcomes included the Expanded Disability Status Scale (EDSS) score and serum 25(OH)D levels.

**Results:**

Nine studies comprising 1,078 participants were included. No significant difference in EDSS scores was observed between the intervention and control groups (mean difference (MD) = −0.11, 95%CI [−0.40, 0.18], *Z* = 0.73, *p* = 0.47). Serum 25(OH)D levels were significantly higher in the intervention group (pooled MD = 44.97, 95%CI [29.93, 60.01], *Z* = 5.86, *p* < 0.001). Subgroup analysis by vitamin D dosage showed that the low/medium-dose group significantly reduced ARR (pooled MD = –0.14, 95%CI [−0.26, −0.03], *p* = 0.01), while the high-dose group had no significant effect on ARR (pooled MD = 0.05, 95%CI [−0.08, 0.18], *p* = 0.42). No significant overall difference in ARR was found between the two groups (pooled MD = -0.07, 95%CI [−0.18,0.03], *Z* = –1.37, *p* = 0.17).

**Conclusion:**

The results of this meta-analysis showed that vitamin D supplementation, regardless of dosage, could significantly increase the serum 25(OH)D level in patients with multiple sclerosis. Subgroup analysis showed that low/medium-dose vitamin D supplementation could significantly reduce the ARR, while high-dose supplementation had no significant effect on ARR. There was no significant difference in EDSS scores between the experimental group and the control group.

## Introduction

Multiple sclerosis (MS) is a chronic autoimmune disorder characterized by inflammatory demyelination within the central nervous system. Its pathological mechanisms involve aberrant immune activation, axonal injury, and disruption of the blood–brain barrier (BBB) (1). Among the various clinical subtypes of MS, relapsing–remitting multiple sclerosis (RRMS) is the most prevalent, accounting for over 80% of all cases (2). The disease course in these patients is marked by recurrent acute relapses, each potentially leading to cumulative neurological deficits, increased disability, and significantly reduced quality of life (3). Consequently, identifying key factors influencing MS relapse and potential interventions remains a major focus in neurology research.

In recent years, the immunomodulatory role of vitamin D in autoimmune diseases has garnered increasing attention, including its association with MS (4). Vitamin D is a fat-soluble vitamin whose active form, 1,25-dihydroxyvitamin D₃ [1,25-(OH)₂D₃], exerts diverse biological effects by binding to the vitamin D receptor (VDR) (5). Immunologically, 1,25-(OH)₂D₃ inhibits the differentiation of T helper 17 (Th17) cells and the release of pro-inflammatory cytokines (e.g., IL-17, TNF-*α*), while promoting the expansion of regulatory T cells (Tregs), thereby maintaining immune homeostasis. From a neuroprotective perspective, it enhances BBB integrity, reduces inflammatory cell infiltration, and mitigates demyelination (6). Based on these mechanisms, vitamin D may theoretically reduce MS relapse risk through immunoregulation and neuroprotection.

However, clinical studies examining the relationship between vitamin D and MS relapse rate have yielded inconsistent results. Some epidemiological studies indicate that low serum 25-hydroxyvitamin D [25(OH)D] levels are associated with an increased risk of MS relapse, whereas interventional trials of vitamin D supplementation have not consistently demonstrated preventive effects (7, 8). These discrepancies may stem from variations in study design, sample size, vitamin D dosage, and follow-up duration. For instance, some small-scale studies may lack statistical power to accurately reflect the true association, and inconsistent definitions and assessment criteria for “relapse” across studies may introduce bias.

Therefore, this study systematically searched published randomized controlled trials (RCTs) and employed meta-analysis to synthesize existing evidence, aiming to clarify the dose–response relationship between serum 25(OH)D levels and the annualized relapse rate (ARR) in MS, and to evaluate the effect of vitamin D supplementation on reducing relapse risk. The findings will provide an evidence-based foundation for the clinical management of MS, facilitate the development of individualized relapse prevention strategies, and ultimately improve long-term patient outcomes.

## Materials and methods

### Inclusion and exclusion criteria

Inclusion Criteria: (1) Study type: RCTs. (2) Participants: Diagnosed with MS according to established criteria (9), aged ≥18 years. (3) Exposure/intervention: Serum 25(OH)D level ≥50 nmol/L. (4) Outcomes: ARR, Expanded Disability Status Scale (EDSS) score, or serum 25(OH)D level. (5) Follow-up duration: ≥6 months.

Exclusion Criteria: (1) Animal studies, reviews, conference abstracts. (2) Patients with other severe neurological disorders. (3) Patients with other autoimmune diseases or receiving specific immunomodulatory therapies. (4) Incomplete or inaccessible data.

### Grouping method

Experimental group: MS patients receiving vitamin D supplementation (including specific doses of vitamin D preparations) or with higher baseline serum 25(OH)D levels.

Control group: MS patients not receiving vitamin D supplementation or with lower baseline serum 25(OH)D levels.

### Definition and assessment of outcome measures

Primary Outcome: ARR was defined as the total number of confirmed relapse events during follow-up divided by the total person-years of follow-up (person-years = sum of individual follow-up durations in years). A “confirmed relapse” was defined as a worsening of neurological function meeting clinical relapse criteria, lasting ≥24 h, excluding reversible factors such as infection or fever, and confirmed by clinical evaluation.

Secondary Outcomes: EDSS score: A quantitative scoring system ranging from 0 to 10, assessing the degree of neurological disability in MS patients. Higher scores indicate more severe disability. The score encompasses eight functional domains: pyramidal, cerebellar, brainstem, sensory, bowel and bladder, visual, cerebral, and other. Assessments were performed by trained neurologists based on clinical examination. Serum 25(OH)D level: Measured using enzyme-linked immunosorbent assay (ELISA) or liquid chromatography–tandem mass spectrometry (LC–MS/MS). Concentrations are reported in nmol/L to assess vitamin D nutritional status.

### Search strategy

Databases: PubMed, Medline, Web of Science, Cochrane Library, EMBASE.

Keywords and Subject Terms: Key terms included: vitamin D 25-hydroxyvitamin D cholecalciferol multiple sclerosis MS relapse recurrence annualized relapse rate ARR. Boolean operators (AND OR) were used to refine the search: (“vitamin D” OR “25-hydroxyvitamin D” OR “cholecalciferol”) AND (“multiple sclerosis” OR “MS”) AND (“relapse” OR “recurrence” OR “annualized relapse rate” OR “ARR”).

Time Range: The search included all relevant studies published from the inception of each database until April 2025 to ensure data timeliness and relevance.

### Literature screening and data extraction

Literature screening was conducted independently by at least two researchers. Initially, clearly irrelevant records were excluded based on title and abstract. Potentially relevant articles were retrieved for full-text evaluation. Extracted data included: basic study information (author, publication year, country), sample size, study type (RCT), baseline characteristics (age, sex, EDSS score), method and level of serum 25(OH)D detection, vitamin D supplementation protocol (dose, duration), follow-up time, and outcome measures (ARR, EDSS score, serum 25(OH)D level). Discrepancies were resolved by a third researcher.

### Quality assessment

The Cochrane Risk of Bias 2 (RoB 2) tool was used to evaluate quality across domains: The assessed domains included random sequence generation (selection bias), allocation concealment (selection bias), blinding of participants and personnel (performance bias), blinding of outcome assessment (detection bias), incomplete outcome data (attrition bias), selective reporting (reporting bias), and other potential biases. Each study was rated as “low risk,” “high risk,” or “unclear.”

### Statistical analysis

RevMan 5.3 was used for analysis. Results are presented as effect estimates with 95% confidence intervals (95%CI). Heterogeneity among included studies was assessed initially, and forest plots and funnel plots were generated. If no significant heterogeneity was observed (*I^2^* < 50%), a fixed-effects model was applied for data synthesis. If moderate or high heterogeneity was present (*I^2^* ≥ 50%), a random-effects model was used. Publication bias was evaluated using funnel plots.

## Results

### Literature search results

**Figure 1:**
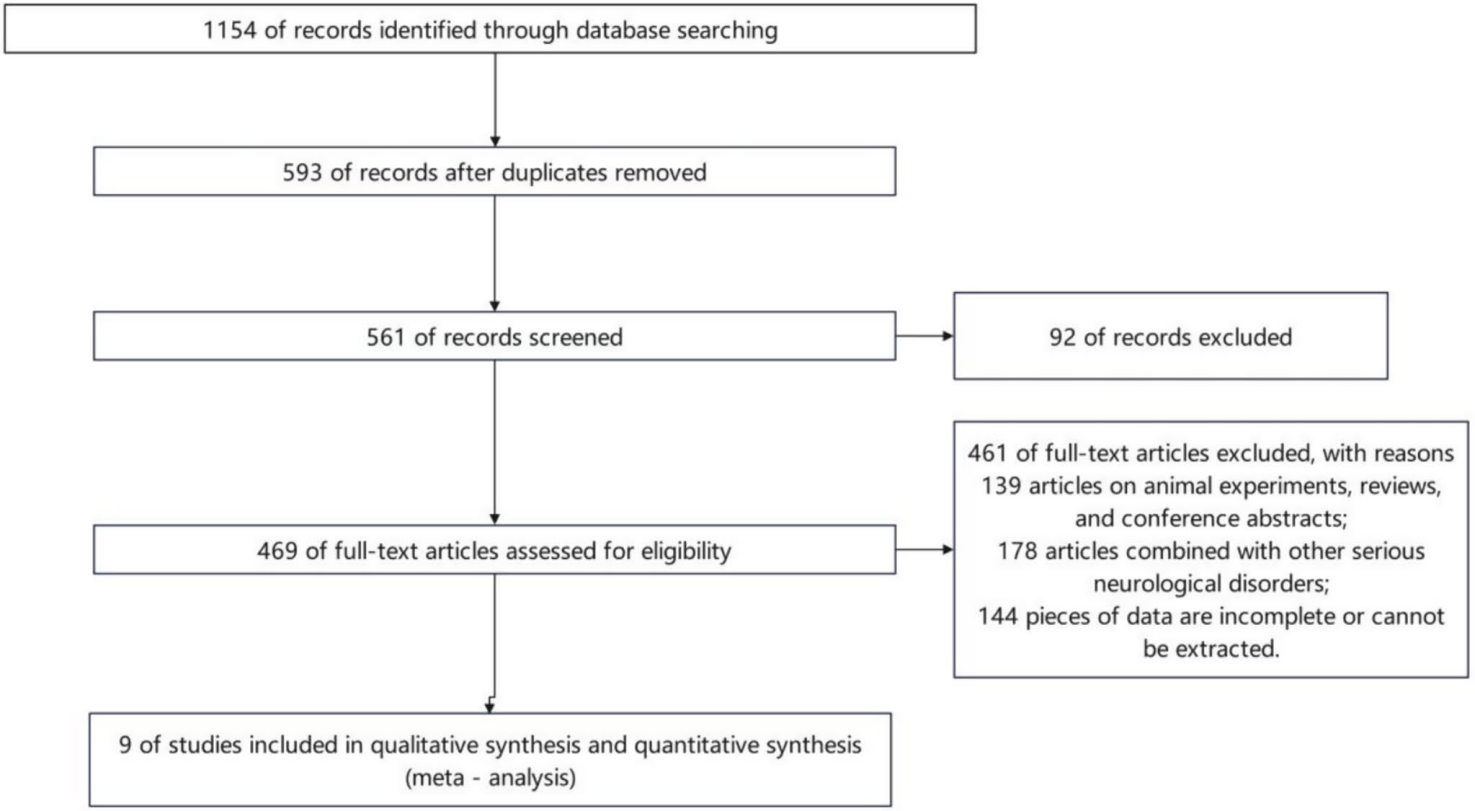
Literature screening flowchart.

A total of 1,154 records were identified through database searching. After removing duplicates, 561 articles remained for full-text assessment. Based on the inclusion and exclusion criteria, 9 studies were ultimately included (Figure 1).

### Characteristics of included studies

A total of 9 RCTs comprising 1,078 participants were included. The basic characteristics of the included studies are presented in Table 1.

**Table 1:** Basic characteristics of included studies.

Included study (Year)	Sample Size (n)	Specific vitamin D dosage	Control group intervention	MS subtype	Disease-modifying therapy use	Follow-up duration (Months)	Baseline ESDD	Percentage of female participants	Drop- out rate	Mean Age (Years)	Mean disease duration (Years)	Outcome measures
Mosayebi (2011) (20)	59	300,000 IU/day	Placebo	RRMS	IFNB-1a	6	Intervention group: Mean 2.1 (range 0–3.5), Placebo group: Mean 2.5 (range 0–3.5)	71.0%	4.8%	36	5.3	EDSS
Ascherio (2014) (21)	468	19-36 nmol/L(Q1), 36-45 nmol/L(Q2), 45-52 nmol/L(Q3), 52-61 nmol/L(Q4), 61.60–98.24 nmol/L(Q5)	Placebo	CIS	IFNB-1b	60	/	71%	0.64%	30.7	/	ARR
Dörr (2020) (22)	53	10,200 IU/day	Low-dose 400 IU/day	RRMS(51), CIS(2)	IFNB-1b	18	High - dose group: Median 2.0 (range 0–5.0), Low - dose group: Median 2.5 (range 0–6.0)	59.8%	22.6%	42.8	9.2	ARR, EDSS, Serum25(OH)D
Kampman (2012) (23)	68	20,000 IU/day	Placebo	RRMS	Interferon beta (31), Glatiramer acetate (2), Natalizumab (1)	24	Intervention group: Median 2.5 (range 0–4.5), Placebo group: Median 2.0 (range 0–4.5)	70.6%	5.6%	40.5	10	ARR, EDSS, Serum25(OH)D
Golan (2013) (24)	45	4,370 IU/day	Low-dose 800 IU/day	RRMS	IFNB-1b	12	Overall median 2.5 (range 0–7.0)	84.4%	33.3%	43.1	6	ARR, EDSS, Serum25(OH)D
Soilu-Hänninen (2012) (25)	66	20,000 IU/day	Placebo	RRMS	IFNB-1b	12	Intervention group: Median 2.0 (range 0–5.0), Placebo group: Median 1.5 (range 0–4.0)	62.1%	6.1%	37	2.7	ARR, EDSS
Burton (2010) (26)	50	28,000–280,000 IU/week(Q1), 10,000 IU/day(Q2), 4,000 IU/day(Q3), 0 IU/day(Q4)	Low-dose ≤4,000 IU/day	RRMS(45), SPMS(5)	IFN(24), GA(4),	12	Treatment group mean: 1.46 (95% confidence interval 0.82–2.10), Control group mean: 1.23 (95% confidence interval 0.54–1.92)	81.6%	8.2%	40.5	7.8	ARR, EDSS
Hupperts (2019) (27)	229	6,670 IU/day(Q1), 14,007 IU/day(Q2)	Placebo	RRMS	IFNB-1a	12	/	67.7%	19.8%	33.8	1.1	ARR
Sotirchos (2016) (28)	40	10,400 IU/day	Low-dose 800 IU/day	RRMS	Interferon beta (6), Glatiramer acetate (10), Natalizumab (11), Fingolimod (4)	6	High - dose group: Median 2.0 (interquartile range 2.0–4.0), Low - dose group: Median 2.5 (interquartile range 2.0–3.5)	70.5%	12.5%	40.0	8	Serum25(OH)D

### Quality assessment

**Figure 2:**
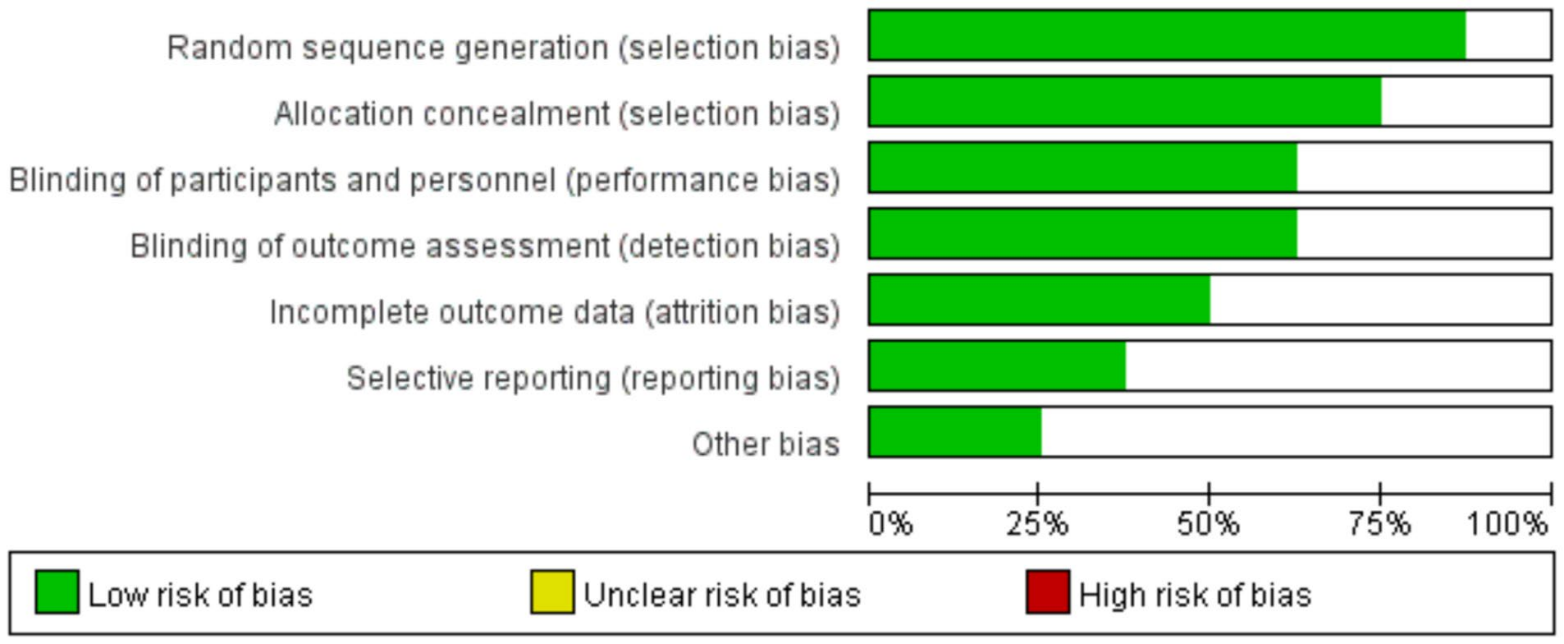
Risk of bias graph.

The methodological quality of the included studies was assessed using the RoB 2 tool for RCTs. The results indicated that some studies had an “unclear risk” in domains such as random sequence generation and allocation concealment. No studies were identified with a definitive “high risk” of bias in any domain. The overall distribution of bias risks was relatively balanced (Figure 2).

### Meta-analysis

**Figure 3:**
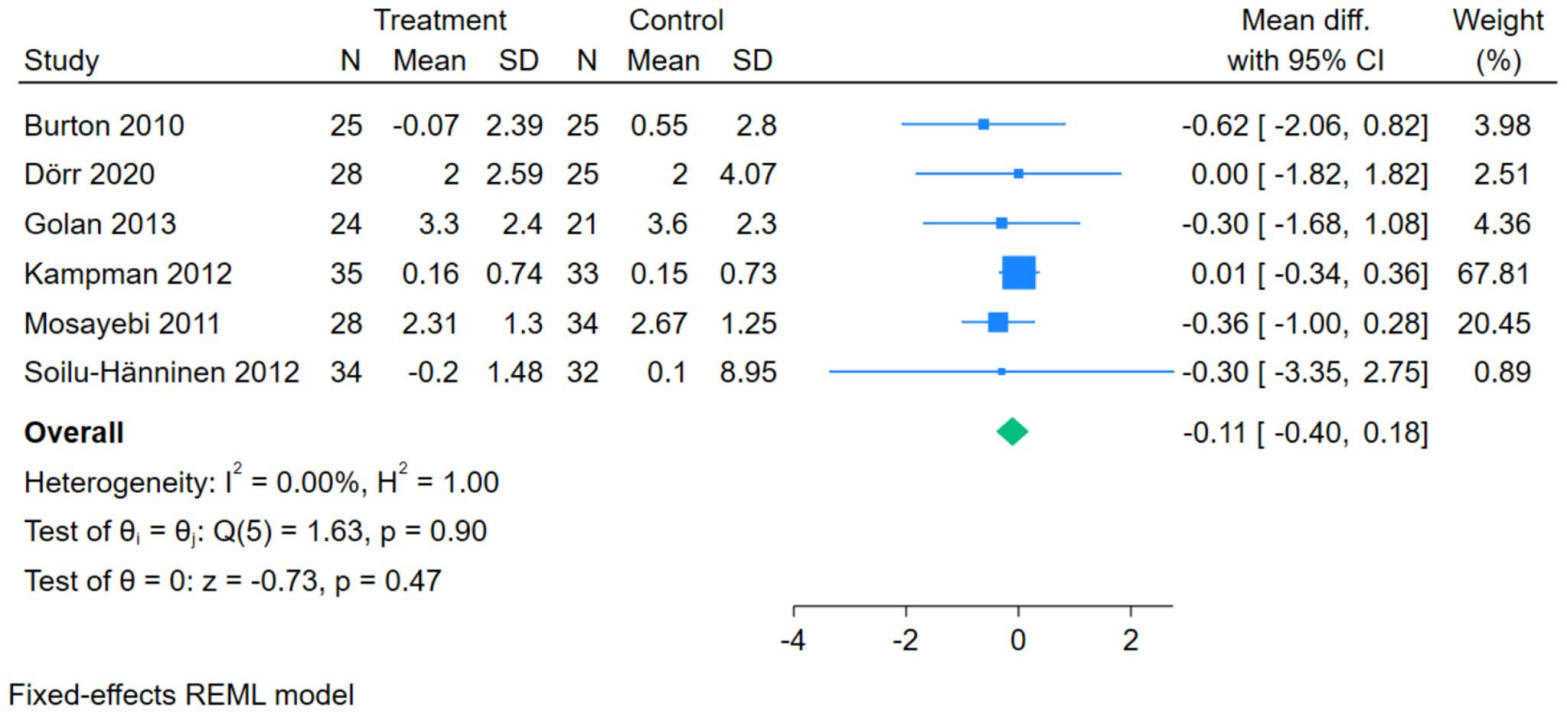
Forest plot of EDSS.

The forest plot for the EDSS compared the association between vitamin D intervention and EDSS scores in 6 studies. The *Q*-test and *I*^2^-test indicated no significant heterogeneity among the studies (*p* = 0.90, Chi^2^ = 1.63, df = 5, *I*^2^ = 0.00%). The mean difference (MD) was calculated using a fixed-effects REML model. The results showed no significant difference in EDSS scores between the treatment and control groups (pooled MD = –0.11, 95%CI [−0.40, 0.18], *Z* = –0.73, *p* = 0.47) (Figure 3).

**Figure 4:**
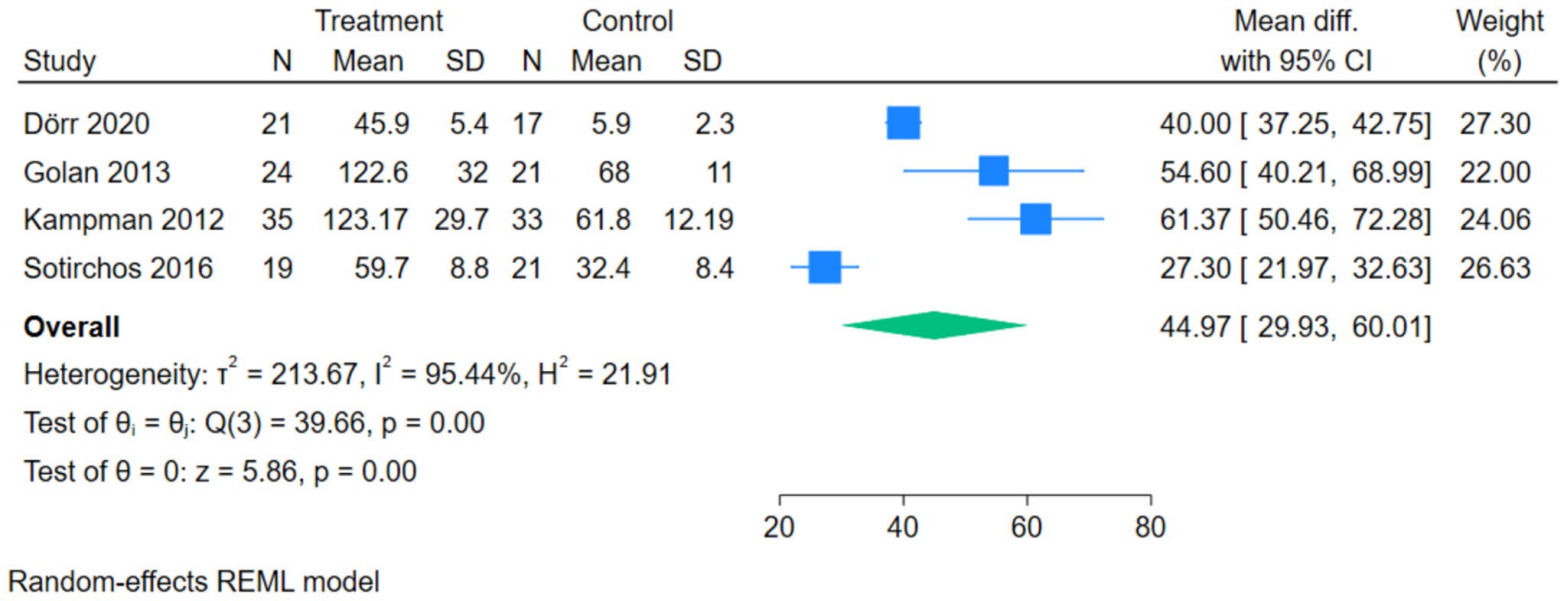
Forest plot of Serum 25(OH)D.

The forest plot of serum 25(OH)D showed that 4 studies compared the correlation between vitamin D intervention and serum 25(OH)D levels in patients with MS. The *Q*-test and *I*^2^-test indicated significant heterogeneity among the studies (*p* = 0.00, Tau^2^ = 213.67, Chi^2^ = 39.66, df = 3, *I*^2^ = 95.44%). The MD was calculated using the random-effects REML model. The results showed a statistically significant difference between the two groups, with the serum 25(OH)D level in the treatment group being significantly higher than that in the control group (pooled MD = 44.97, 95%CI[29.93, 60.01], *Z* = 5.86, *p* = 0.00) (Figure 4).

**Figure 5:**
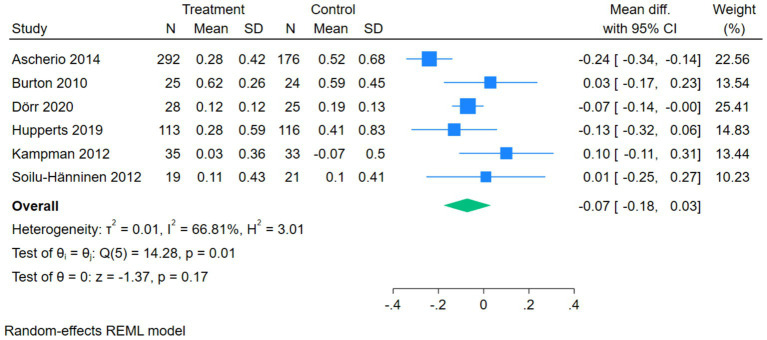
Forest plot of ARR.

The forest plot of ARR showed that 6 studies compared the correlation between vitamin D intervention and ARR in patients with MS. The *Q*-test and *I*^2^-test indicated a certain degree of heterogeneity among the studies (*p* = 0.01, Tau^2^ = 0.01, Chi^2^ = 14.28, df = 5, *I*^2^ = 66.81%). The MD was calculated using the random-effects REML model. The results showed that there was no statistically significant difference between the two groups (pooled MD = –0.07, 95%CI[−0.18,0.03], *Z* = –1.37, *p* = 0.17) (Figure 5).

### Publication bias assessment

**Figure 6:**
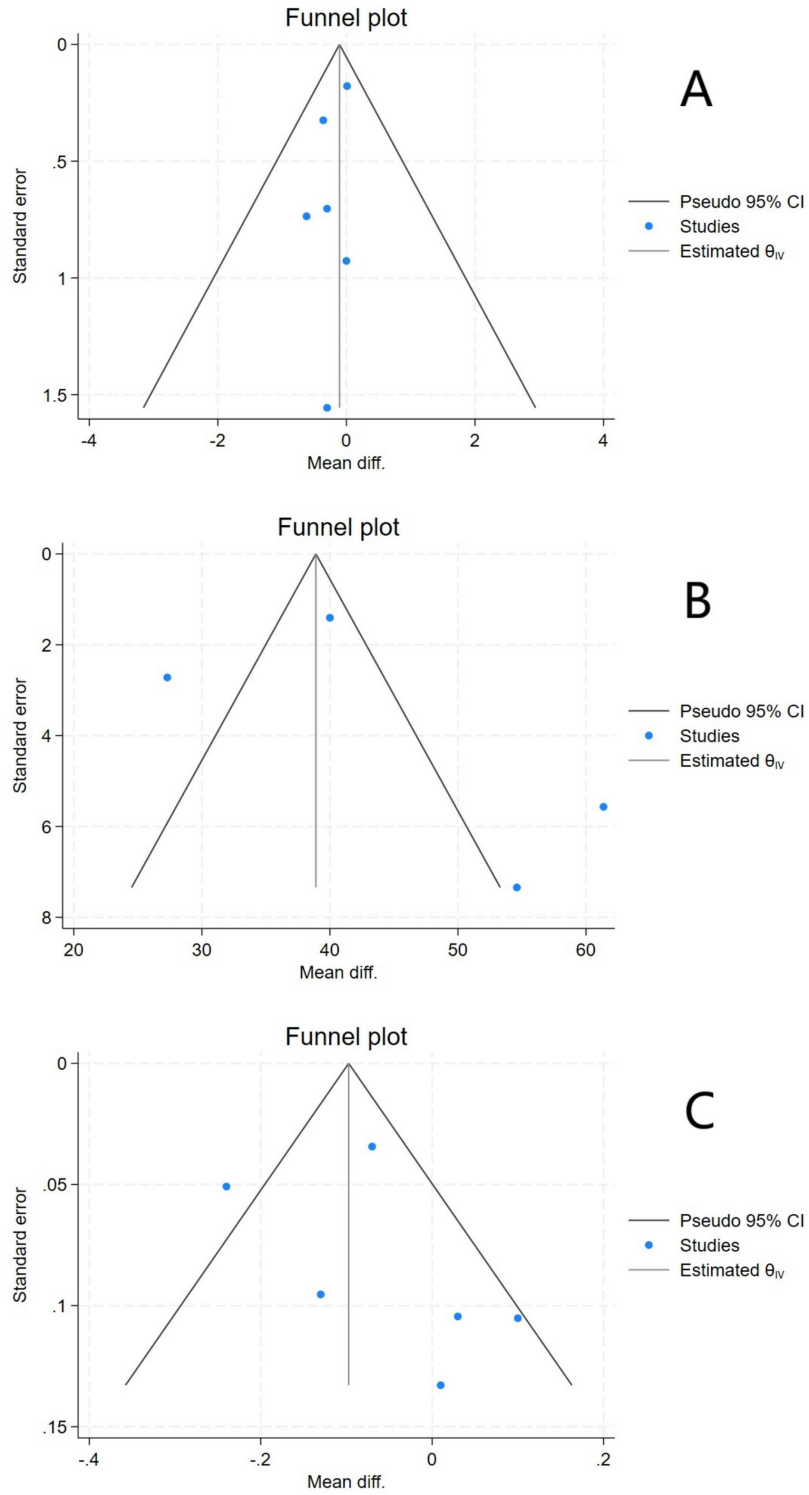
Funnel plots of EDSS **(A)**, Serum 25(OH)D **(B)**, and ARR **(C)**.

The funnel plot for EDSS exhibited suboptimal symmetry, suggesting potential publication bias. The funnel plot for serum 25(OH)D showed poor symmetry, which might be due to the small number of included studies or the presence of publication bias. The funnel plot for ARR demonstrated generally moderate symmetry (Figure 6).

Further Egger’s tests were performed. For EDSS, the Egger’s test result showed *Z* = –0.73, *p* > 0.05, and no significant small sample effect (publication bias) was found. For serum 25(OH)D, the Egger’s test result showed *Z* = 1.55, *p* > 0.05, and no significant publication bias was found. For ARR, the Egger’s test result showed *Z* = 1.29, *p* > 0.05, and no significant small sample effect was found.

### Sensitivity analysis

Sensitivity analysis was conducted by sequentially excluding each included study. For the EDSS sensitivity analysis, *I*^2^ was consistently 0%, and the direction of the pooled effect size remained unchanged during the exclusion process, indicating that the meta-analysis results had good stability. The sensitivity analysis of the serum 25(OH)D forest plot showed that when *Sotirchos 2016* was excluded, the heterogeneity was the lowest (*I*^2^ = 89%), and the direction of the effect size remained unchanged, indicating that it did not affect the overall conclusion. The sensitivity analysis of the ARR forest plot showed that when *Ascherio 2014* was excluded, the heterogeneity changed to *I*^2^ = 0%, and the direction of the effect size remained unchanged, indicating that this study was the main source of heterogeneity.

### GRADE evidence quality evaluation

The GRADE approach (Grading of Recommendations Assessment, Development and Evaluation) was used to evaluate the quality of evidence for each outcome measure (ARR, EDSS score, serum 25(OH)D level). The assessment dimensions included: risk of bias, inconsistency, indirectness, imprecision, and publication bias. Evidence quality was rated as “high,” “moderate,” “low,” or “very low” based on the above dimensions.

(1) Serum 25(OH)D level: Moderate quality evidence. Downgraded once due to high heterogeneity (*I*^2^ = 94%) among included studies. (2) ARR: Low to moderate quality evidence. Downgraded once for moderate heterogeneity (*I*^2^ = 56%) and once for potential publication bias (as suggested by funnel plot asymmetry). (3) EDSS score: Moderate quality evidence. No downgrading was performed, as no significant heterogeneity (*I*^2^ = 0%) or obvious publication bias was observed.

### Subgroup analysis

**Figure 7:**
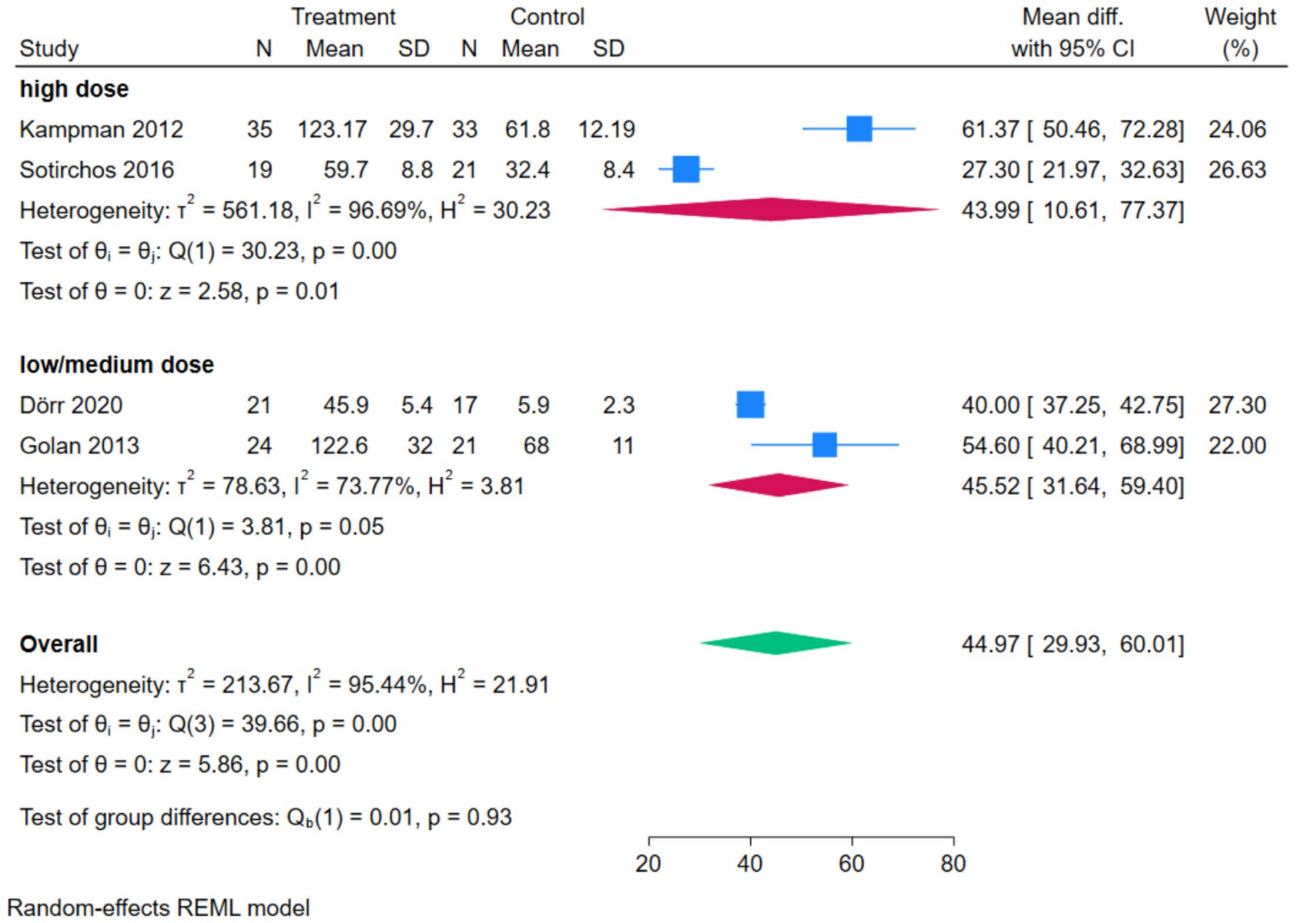
Forest plot of subgroup analysis of serum 25(OH)D.

The subgroup analysis forest plot of serum 25(OH)D was conducted by dividing into a high-dose group and a low/medium-dose group based on vitamin D dosage. The high-dose group included 2 studies, with a MD of 43.99 (95%CI [10.61, 77.37]), *Z* = 2.58, *p* = 0.01, suggesting that high-dose vitamin D intervention could significantly increase serum 25(OH)D levels, while there was high heterogeneity within the subgroup (*I*^2^ = 96.69%, *p* = 0.00). The low/medium-dose group included 2 studies, with a pooled MD of 45.52 (95%CI [31.64, 59.40]), *Z* = 6.43, *p* = 0.00, indicating that low/medium-dose intervention could also significantly increase serum 25(OH)D levels, and there was a certain degree of heterogeneity within the subgroup (*I*^2^ = 73.77%, *p* = 0.05). The overall pooled MD was 44.97 (95%CI [29.93, 60.01]), *Z* = 5.86, *p* = 0.00, indicating that regardless of the dosage, vitamin D intervention could significantly increase serum 25(OH)D levels. The test for subgroup differences showed Qb(1) = 0.01, *p* = 0.93, suggesting that there was no statistically significant difference in effect size between the high-dose group and the low/medium-dose group (Figure 7).

**Figure 8:**
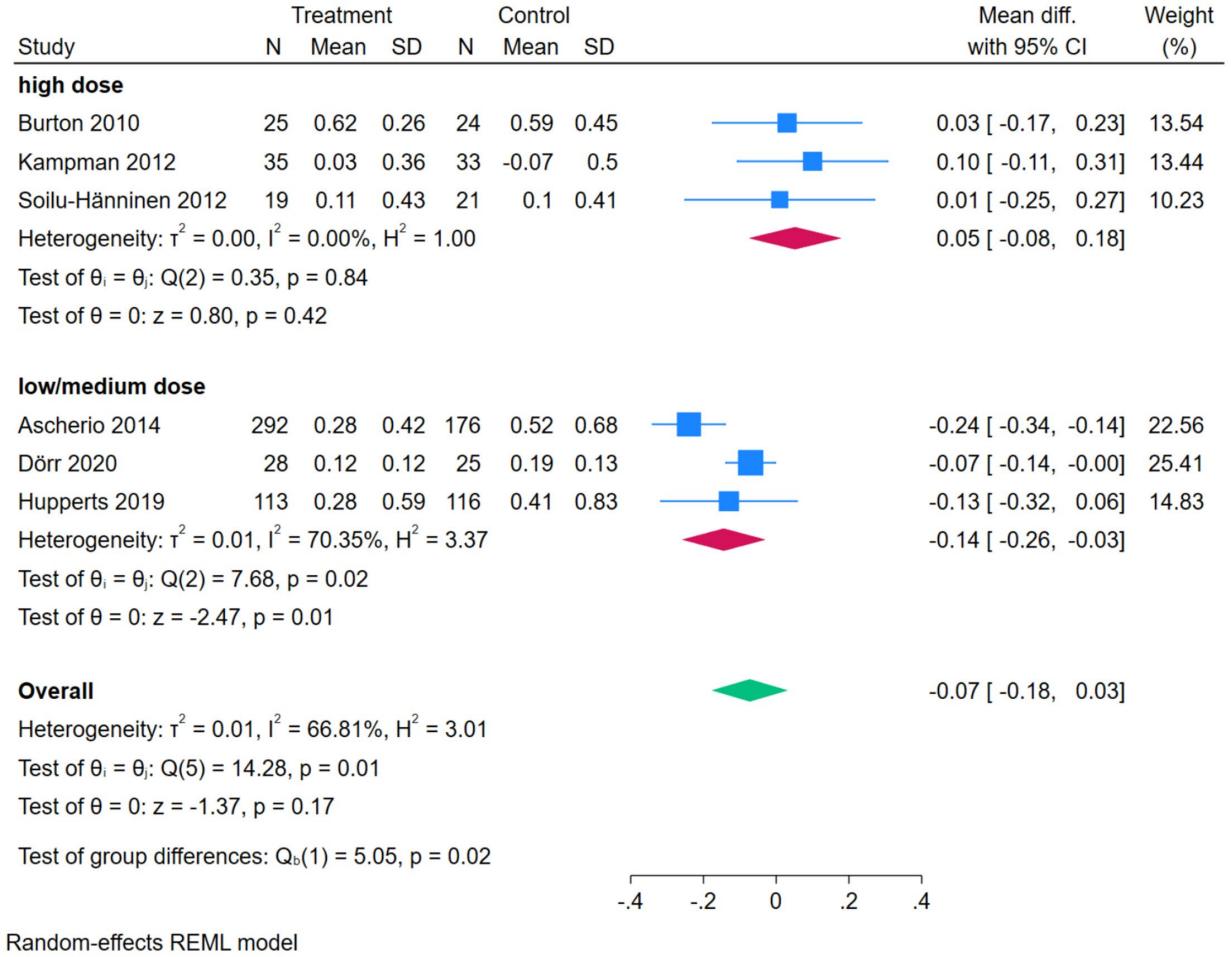
Forest plot of subgroup analysis of ARR.

The subgroup analysis forest plot of ARR was conducted by dividing into a high-dose group and a low/medium-dose group based on vitamin D dosage. The high-dose group included 3 studies, with extremely low heterogeneity (*I*^2^ = 0.00%), and an MD of 0.05 (95%CI [−0.08, 0.18], *p* = 0.42), showing no significant effect on ARR. The low/medium-dose group included 3 studies, with a certain degree of heterogeneity (*I*^2^ = 70.35%), and a pooled MD of −0.14 (95%CI [−0.26, −0.03], *p* = 0.01), which could significantly reduce ARR. The overall pooled MD was −0.07 (*p* = 0.17), showing no significant overall difference, but the test for subgroup differences (Qb = 5.05, *p* = 0.02) suggested that there was a statistically significant difference in effect between different dosage groups, and dosage was one of the sources of heterogeneity (Figure 8).

## Discussion

Multiple sclerosis is an autoimmune disorder characterized by central nervous system demyelination. The disease progression in patients with RRMS is closely associated with recurrent acute attacks, each of which may lead to irreversible neurological damage and disability accumulation (10, 11). Vitamin D, a fat-soluble vitamin with both immunomodulatory and neuroprotective properties, has been extensively studied regarding its association with MS relapse risk (12). This meta-analysis, integrating data from 1,078 patients across nine high-quality studies, demonstrated that vitamin D supplementation (regardless of dosage) significantly increased serum 25(OH)D levels. Subgroup analysis by dosage showed that low/medium-dose vitamin D supplementation could significantly reduce ARR, while high-dose supplementation had no significant effect on ARR. Additionally, vitamin D supplementation did not significantly affect the EDSS score. These findings provide important evidence for understanding the role of vitamin D in MS management and highlight the importance of dosage optimization. The following discussion integrates potential mechanisms and clinical implications.

The results of this study showed that the serum 25(OH)D level in the experimental group was significantly higher than that in the control group (pooled MD = 44.97, 95%CI [29.93, 60.01], *Z* = 5.86, *p* < 0.001), and subgroup analysis confirmed that both high-dose and low/medium-dose vitamin D supplementation could achieve this effect, with no significant difference in efficacy between the two dosage groups. This finding suggests that low vitamin D levels are closely associated with an increased risk of MS onset and higher disease activity, and vitamin D supplementation can effectively improve vitamin D nutritional status regardless of dosage. The biological mechanism underlying this effect is the pleiotropic action of vitamin D: its active form, 1,25-(OH)₂D₃, regulates the immune system via the VDR, inhibits Th17 differentiation and the release of pro-inflammatory cytokines (e.g., IL-17, TNF-*α*), and promotes regulatory Treg proliferation, thereby mitigating autoimmune attacks on the central nervous system (13, 14). Notably, the dosage-dependent effect on ARR may be related to the saturation of vitamin D’s biological effects: low/medium doses may reach the optimal concentration for immunomodulation, effectively suppressing acute inflammatory responses and reducing relapse risk. While excessively high doses may not further enhance immune regulation, or even lead to potential adverse effects that offset the protective effect, resulting in no significant reduction in ARR. Furthermore, 1,25-(OH)₂D₃ enhances BBB integrity, reducing inflammatory cell infiltration and demyelinating damage, which further decreases relapse risk. From a clinical perspective, the reduction in ARR is meaningful (15, 16). As a key indicator for assessing the disease activity of MS, the ARR directly reflects the effect of intervention measures on reducing the risk of relapse and serves as an important basis for judging treatment effectiveness in clinical decision-making. In addition, there are gender-dependent structural and functional differences in immune cells, and we found that the percentage of female subjects in each study was higher than that of male subjects (17).

However, the results of this study showed that there were no significant differences in ARR or EDSS scores between the experimental group and the control group. This does not negate the value of vitamin D but rather reflects the complexity of MS progression. The EDSS primarily assesses irreversible neurological disability, which is influenced by long-term pathological processes such as axonal loss and glial scar formation. In contrast, the protective effects of vitamin D may be more focused on suppressing acute inflammation and reducing new lesions, with limited impact on pre-existing disability (18). Additionally, the follow-up duration across the included studies (ranging from 6 months to 3 years) may have been insufficient to detect significant changes in ARR and EDSS. This suggests that the neuroprotective effects of vitamin D may be time-dependent and stage-specific. Clinical evaluation of its impact on disability progression should consider the disease course and avoid relying solely on short-term follow-up data.

This study adhered strictly to the preferred reporting standards for systematic reviews and meta-analyses. A comprehensive search was conducted across five major databases (PubMed, Medline, Web of Science, Cochrane Library, and EMBASE). Eight high-quality RCTs comprising 1,038 patients were included, ensuring representativeness and statistical power. The RoB-2 tool was used for quality assessment, and rigorous bias control was implemented.

The findings are consistent with most previous meta-analyses. A meta-analysis focused on high-dose vitamin D3 (≥1,000 IU/day) as adjuvant treatment for MS, including 867 participants with outcomes centered on the EDSS, ARR, and new T2 lesions (19). The conclusion was that there was no significant effect on clinical outcomes, with only a non-significant trend of reduction in new T2 lesions. However, this study did not conduct dosage-stratified analysis and thus failed to identify differences in the effects of vitamin D at different dosages. In contrast, our study included 1,038 participants, and subgroup analysis confirmed that low/medium-dose vitamin D could significantly reduce ARR. Additionally, regardless of dosage, vitamin D supplementation significantly increased serum 25(OH)D levels. We also systematically evaluated the associations between EDSS, ARR, and vitamin D nutritional status, placing greater emphasis on the dose-effect relationship and the individualized impact of vitamin D on MS relapse.

Based on these findings, vitamin D supplementation (preferably low/medium dose) may be recommended as an important adjunct strategy for preventing MS relapses. Specific recommendations include: regular monitoring of serum 25(OH)D levels in all MS patients. Initiation of low/medium-dose supplementation in those with levels below 50 nmol/L, with a target range of 75–125 nmol/L (avoiding excessive high-dose supplementation unless clinically justified). use of vitamin D as an adjunct to disease-modifying therapies (DMTs), especially in patients with suboptimal response or intolerance to DMTs. and individualization of dosage based on patient characteristics (e.g., baseline 25(OH)D level, disease activity, comorbidities) to maximize efficacy. Given the delayed response in EDSS and the dosage-dependent effect on ARR, long-term follow-up of at least 5 years is recommended to evaluate the sustained impact of vitamin D on disability progression and optimize supplementation protocols.

Future research should focus on: conducting multicenter, large-sample RCTs to compare the effects of different vitamin D doses on relapse rate and long-term disability. Investigating the synergistic mechanisms between vitamin D and DMTs. and integrating genomic approaches to identify biomarkers associated with vitamin D response for personalized intervention.

In conclusion, this meta-analysis confirms that vitamin D supplementation significantly increases serum 25(OH)D levels, but does not significantly improve ARR and EDSS scores. These results provide evidence supporting the use of vitamin D supplementation for relapse prevention in clinical practice, while also highlighting the importance of long-term follow-up and individualized strategies. Further research is needed to elucidate the underlying mechanisms and optimize interventional protocols for improving outcomes in MS patients.

## Data Availability

The original contributions presented in the study are included in the article/supplementary material, further inquiries can be directed to the corresponding author/s.
